# Pronounced temporal velocity variations within the fault fracture zone in response to Earth tide modes

**DOI:** 10.1093/nsr/nwaf023

**Published:** 2025-01-22

**Authors:** Tenghui Sun, Huajian Yao, Hongfeng Yang, Chang Yu, Song Luo, Yixiao Sheng

**Affiliations:** Laboratory of Seismology and Physics of the Earth's Interior, School of Earth and Space Sciences, University of Science and Technology of China, Hefei 230026, China; Hefei National Laboratory, Hefei 230094, China; Laboratory of Seismology and Physics of the Earth's Interior, School of Earth and Space Sciences, University of Science and Technology of China, Hefei 230026, China; Hefei National Laboratory, Hefei 230094, China; Mengcheng National Geophysical Observatory, University of Science and Technology of China, Mengcheng 233500, China; CAS Center for Excellence in Comparative Planetology, University of Science and Technology of China, Hefei 230000, China; Department of Earth and Environmental Sciences, Faculty of Science, The Chinese University of Hong Kong, Hong Kong 999077, China; Shenzhen Research Institute, The Chinese University of Hong Kong, Shenzhen 518000, China; Laboratory of Seismology and Physics of the Earth's Interior, School of Earth and Space Sciences, University of Science and Technology of China, Hefei 230026, China; Laboratory of Seismology and Physics of the Earth's Interior, School of Earth and Space Sciences, University of Science and Technology of China, Hefei 230026, China; Laboratory of Seismology and Physics of the Earth's Interior, School of Earth and Space Sciences, University of Science and Technology of China, Hefei 230026, China

**Keywords:** seismic velocity variations, fault monitoring, Earth tides, fault fracture zone

## Abstract

Continuous monitoring of seismogenic faults can advance our understanding of the evolution process, holding important keys for forecasting future earthquakes. We report here seismic velocity variations around the Anninghe fault zone in southwest China based on seismic interferometry techniques. We observed that tidal forces significantly impact velocity changes within the fault fracture zone, inducing periodic changes in seismic velocity on diurnal, semidiurnal and monthly scales. Moreover, the response to Earth tides is notably more pronounced in the fault fracture zone compared to other areas. This can be attributed to tidal forces affecting the opening and closing of cracks in the subsurface medium. Due to the higher density of fractures within the fault fracture zone, it becomes more sensitive to tidal forces. Our findings underscore the crucial role of tidal forces in perturbing stress within the fault zone during periods when earthquakes have not occurred.

## Teaser

Tidal forces induce significant seismic velocity changes in the Anninghe fault zone, offering new insights into their role in stress perturbations within fault fracture zones.

## INTRODUCTION

The stress state within a fault zone is crucial to the occurrence of earthquakes [1–4]. Investigation of the stress state and its evolution may help identify precursors to earthquakes [5] and understand the mechanisms in various tectonic and non-tectonic processes. However, it remains challenging to measure the stress *in-situ*, and most studies are relying on indirect evidence, such as hydraulic response [6], earthquake focal mechanism [7] or subsurface velocity changes [8,9]. Researchers usually conduct continuous monitoring of seismic velocity changes through seismic ambient noise, and they have achieved significant progress in detecting seismic velocity changes resulting from earthquakes in fault zones using this method [10–12].

**Figure 1.**
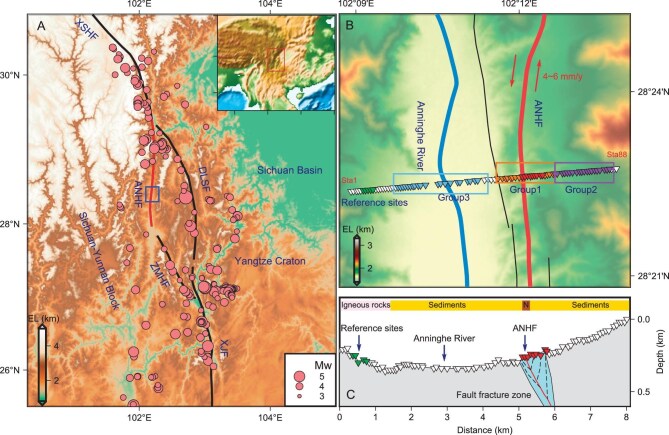
Tectonic and station distribution maps of the Anninghe fault zone and its surrounding areas. (A) The distributions of tectonic units and earthquakes. The main blocks include the Sichuan–Yunnan Block, the Sichuan Basin and the Yangtze Craton. The main faults include the Xianshuihe fault (XSHF), the Anninghe fault (ANHF), the Daliangshan fault (DLSF), the Zemuhe fault (ZMHF) and the Xiaojiang fault (XJF). The pink dots show earthquakes near the faults from 1970 to 2012 with a magnitude greater than 3.0. The blue rectangle represents the study region shown as (B). (B) The triangles indicate positions of the seismometers. The station numbers range from Sta1 to Sta88, from west to east, totaling 88 stations. Orange triangles represent Group 1 stations situated in proximity to the fault zone. Purple and light blue triangles represent Group 2 and Group 3 stations, respectively, located farther from the fault zone. Red and black lines show the two branches of the Anninghe fault and the eastern branch (thick red line) is more active. The arrows show the slip direction of the strike-slip fault. Blue line represents the Anninghe River. The light green triangles indicate the reference sites used for calculating the standard spectral ratio. (C) A schematic illustration depicting the fault fracture zone and the associated local geological maps (‘N’ represents Neogene) near the stations.

Although many cases of velocity changes shortly before and after large earthquakes have been reported [8,10], changes during the interseismic period are usually small in magnitude and thus have so far received little attention. Furthermore, various environmental factors, including temperature, pressure and precipitation, can have an impact on seismic wave velocity [13–17]. These factors can introduce uncertainties and challenges in studying stress changes within fault zones. Currently, there is a lack of effective methods to significantly reduce the influence of these environmental factors on seismic velocity measurements of fault zones. Here, we derive velocity changes and infer stress responses by applying the wavelet method, which enables the measurement of tiny relative perturbations (on an order of ∼10^−4^) in seismic velocity [18], to continuous ambient noise data recorded by a dense array across the Anninghe fault in the southeastern margin of the Tibetan Plateau, southwest China (Fig. 1A).

The Anninghe fault zone is a significant north-south left-lateral strike-slip fault and exhibits strong tectonic activity, with a horizontal strike-slip rate ranging from 4 to 6 mm/year [19]. Historically there have been multiple earthquakes with magnitudes greater than M7 [20]. Multiple studies conducted from the perspectives of geology, geophysics, and geochemistry consistently indicate that the Anninghe fault has a significant potential for a major earthquake [19–22]. At present it is considered as a seismic gap that has accumulated high levels of stress and no earthquakes with a magnitude greater than M4 have been recorded in this region over the past 30 years [20]. Investigating the changes in strain state within the Anninghe fault zone by monitoring seismic velocity changes is crucial for studying fault stability and earthquake triggering mechanisms.

Earth tides refer to the deformation of the Earth caused by the gravitational forces exerted by the Moon, Sun and other celestial bodies. The study of Earth tides is of significant importance in understanding various phenomena on our planet. Theoretical investigations have indicated that Earth tides have the potential to influence the stress state of fault zones [23]. Furthermore, numerous studies have provided evidence that tidal forces can play a role in modulating the occurrence of earthquakes [23–26], although controversial debates still exist [27,28]. However, there is still no high-resolution observation on how seismic velocity within the fault zone responds to stress changes associated with tidal forces.

## RESULTS

### Temporal velocity variations from ambient noise

**Figure 2.**
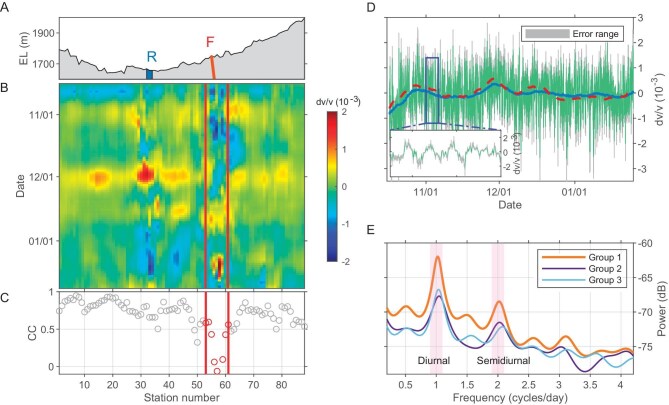
The relative seismic velocity changes (dv/v) at daily and hourly resolution. (A) The spatial distribution of river and fault zone. The blue area represents the Anninghe River (‘R’) and the red line represents the Anninghe fault (‘F’). (B) The daily resolution dv/v of each station in the frequency band of 1–2 Hz. The red lines represent the fault fracture zone. (C) The correlation coefficient between the dv/v curve of each station and the average dv/v curve of all stations in Fig. 2B. The red dots correspond to the red triangles in Fig. 1B and C, which represent the correlation coefficients of stations within the fault fracture zone. (D) Average hourly resolution dv/v for Group 1, Group 2 and Group 3 in the frequency bands of 1–2 Hz. The green line represents the dv/v values, with the gray area indicating the error margin. The blue line illustrates the long-period component of the hourly resolution dv/v, while the red dashed line represents the average daily resolution dv/v for the three station groups. The inset figure provides a zoomed-in view of a 5-day time window starting on November 1. (E) Diurnal and semidiurnal peaks in the spectra of hourly resolution dv/v for the three station groups.

**Figure 3.**
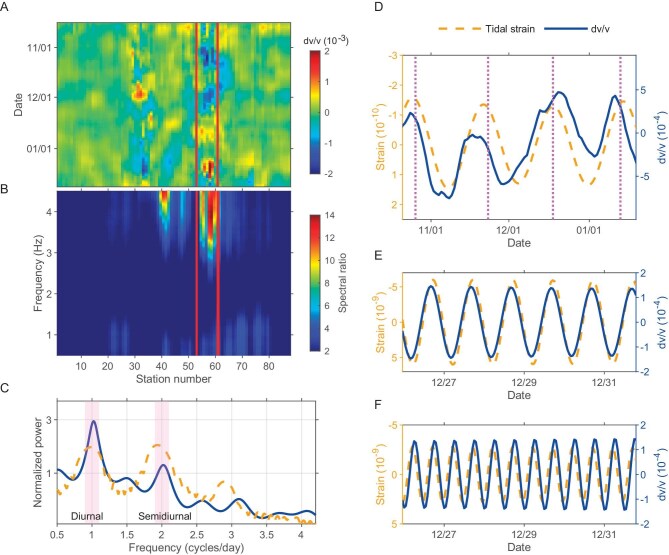
(A) The daily resolution relative seismic velocity changes (dv/v) after removing the influence of environmental factors in the frequency band of 1–2 Hz. The location of the fault fracture zone is denoted by the red lines. (B) The average spectral ratio results along the array obtained from seismic waves of 13 teleseismic earthquakes. The location of the fault fracture zone is denoted by red lines. (C) Comparison of the spectra of dv/v at hourly resolution of Group 1 stations and the tidal strain in the vertical component. The blue solid line represents the dv/v, the yellow dashed line represents the tidal strain, and the pink shadows indicate diurnal and semidiurnal periods. (D) Comparison of the average dv/v of stations within the fault fracture zone and monthly vertical tidal strain time series. The purple dashed line marks the perigee date. (E) Comparison of diurnal dv/v time series for Group 1 stations with vertical tidal strain. (F) Comparison of semidiurnal dv/v time series for Group 1 stations with vertical tidal strain. Tidal strain positive values indicate expansion, while negative values indicate compression.

In this study, we computed seismic velocity variations based on continuous ambient noise in the vicinity of the Anninghe fault zone (Fig. 1B and C). We utilized the wavelet method [18] to analyze relative seismic velocity changes (dv/v) using ambient noise data collected from the linear dense array of 88 stations. These stations were positioned perpendicular to the Anninghe fault zone (Fig. 1B) and spaced ∼50–100 meters apart. The data were recorded over a period of 100 days. This method utilizes the coda wave of auto-correlation function to calculate dv/v, which is less sensitive to directional changes in noise source distribution compared to direct waves [29]. To investigate the spatial and temporal patterns of dv/v variations within the study area, we calculated the daily resolution dv/v for each station and categorized them into two distinct frequency bands: 1–2 Hz (Fig. 2B) and 2–3 Hz (Fig. S1B) (see the Method section for more details). Furthermore, to highlight the differences in dv/v among stations, we computed the correlation coefficient between the dv/v curve of each station and the average dv/v curve of all stations (Fig. 2C and Fig. S1C). To investigate the characteristics of short-period seismic velocity variations, we selected three distinct groups of 21 stations each (Fig. 1B) and computed the hourly resolution dv/v for all groups (Fig. S2A–C) as well as the average dv/v across all three groups (Fig. 2D). Spectrogram analysis of the dv/v for all groups revealed prominent high-energy peaks at diurnal (1 cycle per day) and semidiurnal (2 cycles per day) frequencies (Fig. 2E and Fig. S2D–F). To further analyze these patterns, we applied a filtering method to the hourly resolution dv/v (Fig. S2A) from Group 1 stations to extract the time series for the diurnal and semidiurnal periods (Fig. 3E and F).

We compared the long-period components of hourly resolution dv/v and daily resolution dv/v and found consistent results (Fig. 2D), demonstrating the reliability of both processing methods. Daily resolution results are more effective for analyzing the spatial distribution characteristics of dv/v (Fig. 2B and C), whereas hourly resolution results are better suited for examining temporal variation patterns (Fig. 2E). Combining these two approaches enhances our ability to analyze the spatiotemporal characteristics of dv/v.

In the coda wave window, before reaching six mean free times, waves are predominantly surface waves and are more sensitive to shallow changes [30]. The mean free time is calculated as t = *l*/*c*, where *l* is the transport mean free path and *c* is the energy velocity [30,31]. In this study, we estimated *l* = 72 km and *c* = 3.4 km/s, giving six mean free times of 127 s (Text S1) [32,33]. We use the coda (with arrival time between 30 to 60 s), mainly consisting of scattered surface-wave content. To assess the depth sensitivity of dv/v, we employed Rayleigh wave kernels in conjunction with a 400 m vertical-resolution velocity model of the Anninghe fault zone [34]. The dv/v measurements within the 1–2 Hz and 2–3 Hz frequency ranges exhibited the highest sensitivity to the subsurface medium at depths of ∼700 m and 400 m, respectively (Fig. S3).

### Analysis of the tidal strain and local site effects

To analyze the factors contributing to velocity changes, we calculated the theoretical tidal strain induced by tidal forces and applied the Standard Spectral Ratio (SSR) method. The SSR method, a well-established empirical technique for evaluating local site effects, involves selecting a bedrock outcrop as the reference site. The spectral ratios between the sites under investigation and this reference site provide frequency-dependent amplification factors influenced by local site conditions [35]. First, we employed the PyGTide program [36] to calculate the theoretical tidal strain in the study area. Here we only analyzed the vertical strain because the dv/v results from vertical component correlation functions are mainly due to velocity changes of Rayleigh waves, which are mostly sensitive to vertically polarized shear wave speeds. We conducted spectral analysis on the vertical strain results, extracting time series corresponding to monthly, diurnal and semidiurnal periods (Fig. S4). Both the power spectrum and these time series were then compared with the dv/v results (Fig. 3C–F). Furthermore, the SSR analysis revealed that the horizontal ground motions at stations around the Anninghe fault zone were greater (Fig. 3B), and we identified the area with SSR anomalies as the location of the fault fracture zone.

## DISCUSSION

Previous studies have shown that precipitation, air temperature and barometric pressure are factors that influence seismic velocity changes in the subsurface [13–17]. Considering our array aperture (8 km), it is expected that all stations would experience similar environmental influences (defined here as precipitation, air temperature and barometric pressure). In the daily resolution dv/v analysis, we observed high consistency among the dv/v values of most stations that exhibited high correlation coefficients (Fig. 2C and Fig. S1C), which indicates that similar environmental influences lead to similar dv/v. The consistent velocity variation characteristics enhance the reliability of our findings. However, stations in areas affected by river (Fig. S1C) and fault zone (Fig. 2C and Fig. S1C) exhibit lower correlation coefficients. This could be attributed to the influence of local geological features, such as changes in subsurface structure or properties, which can contribute to the observed deviations from the overall trend.

We hypothesize that the dv/v within the fault fracture zone is influenced by both environmental factors and fault characteristics, while in other areas the dv/v is primarily driven by environmental factors. In order to gain a clearer understanding of the dv/v within the fault fracture zone, we need to isolate the influence of environmental factors. Given the challenges in quantifying the theoretical effect of environmental factors on velocity changes, we use a practical approach to discern their individual contributions. Specifically, we computed the average dv/v from stations with a strong correlation (correlation coefficient >0.75, Fig. 2C), capturing dv/v predominantly attributed to environmental factors. Upon observation of the extracted dv/v, it was found that the results in the 2–3 Hz range are primarily influenced by temperature variations (Fig. S5). Temperature fields induce thermoelastic strain, which in turn causes changes in seismic velocity. Although the temperature changes themselves penetrate only tens to hundreds of centimeters into the crust, the resulting thermoelastic strain can extend much deeper [14,37]. In contrast, the results in the 1–2 Hz range do not exhibit a distinct dominant factor and are likely the outcome of multiple factors influencing the changes. Subsequently, we subtracted the influence of environmental factors from the overall dv/v (Fig. S6). Interestingly, after removing the environmental influence, significant velocity changes are still evident within the fault fracture zone (Fig. 3A). Moreover, the average remaining dv/v within the fault fracture zone exhibits distinct monthly fluctuations (Fig. 3D). It is worth noting that some environmental influence may not have been fully eliminated, as dv/v responses can vary due to differences in medium properties along the 8-km profile [38]. Since barometric pressure, temperature, and precipitation do not exhibit monthly periodic changes (Fig. S7), we believe that our method has effectively minimized the impact of most environmental factors. Therefore, the remaining monthly periodic velocity changes are likely influenced by other factors.

Considering the ∼1-month period of the lunar orbit around the Earth, we propose that the monthly variations in seismic velocity are attributed to the influence of tidal forces. We compared this monthly component tidal strain generated by tidal forces (Fig. S4C) with the dv/v within the fault fracture zone. The result revealed a strong correlation among the dv/v, the tidal strain and the position of the Moon in orbit (Fig. 3D). Moreover, the observation of this phenomenon solely within the fault fracture zone suggests that the fault fracture zone has an increased sensitivity to tidal effects.

In our analysis of the hourly resolution dv/v, we have also observed characteristics that indicate the presence of tidal effects. We analyze the hourly resolution dv/v in the frequency domain, which helps to mitigate the influence of long-period environmental factors [39]. The consistent dv/v characteristics observed across three independent station groups (Fig. 1B and Fig. 2E) provide credibility to our findings. When comparing the spectrum of dv/v with that of tidal strain, we observed that the dv/v spectrum displays two prominent peaks at diurnal and semidiurnal frequencies, mirroring the peaks found in the tidal strain spectrum (Fig. 3C). This synchronization is likely to occur when dv/v is predominantly influenced by Earth tides and when the relaxation time of dv/v in response to strain changes is considerably shorter than the semidiurnal period. However, we observed that the diurnal component of the velocity variation is stronger than the semidiurnal component, in contrast to the Earth tide signal where the semidiurnal component is typically stronger. We attribute this disparity to the diurnal cycle variation of temperature. It is possible that temperature and Earth tides jointly influence the velocity variation, resulting in a stronger diurnal cycle component of the velocity variation [39]. In addition, while all station groups observed diurnal and semidiurnal periodic velocity changes, Group 1—located closer to the fault fracture zone (Fig. 1B)—demonstrated more pronounced peak energy in their velocity variation measurements (Fig. 2E). Since all stations experienced similar environmental influences, this observation highlights that, beyond environmental factors, Earth tides significantly impact the diurnal and semidiurnal velocity changes. Moreover, the time series for diurnal and semidiurnal periods of dv/v align well with the time series of tidal strain (Fig. 3E and F).

**Figure 4.**
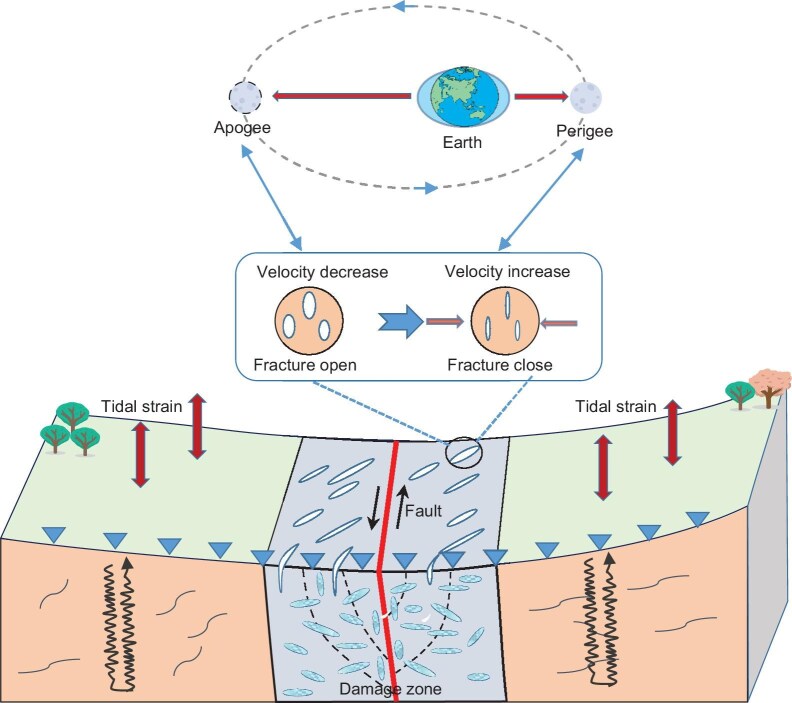
The schematic diagram depicts how tidal strain impacts seismic velocity changes within the fault zone. The fracture zone around the Anninghe fault contains multiple fractures [45], and the opening and closing of these fractures due to tidal strain can modulate relative seismic velocity changes (dv/v).

The key question now is how the Earth tides influence the dv/v within the Anninghe fault zone and why the fault fracture zone exhibits an amplified response to tidal influences. Typically, in a fault fracture zone, most of the slip and deformation occurs within a narrow fault core. However, there is also a broader region surrounding the fault, known as the fault fracture zone. This zone extends beyond the immediate fault core and is characterized by the presence of extensive cracks, fractures and damaged rocks [40–42]. Notably, in the Anninghe fault zone, the velocity model indicates the presence of low-velocity anomalies [34]. Additionally, the standard spectral ratio results reveal more pronounced ground motions (Fig. 3B). This enhanced ground motion is interpreted as the consequence of trapped waves within the highly fractured, lower-velocity materials that constitute the fault zone [43,44]. The measurement of tectonic discontinuities in the outcrops reveals that the number of fractures in the fault fracture zone is nearly ten times greater than in the wall rock [45]. These combined observations strongly suggest the presence of a more fractured medium within the Anninghe fault zone. Tidal forces can affect velocity changes by generating tidal strain, which promotes the opening and closing of subsurface cracks [46–48]. When tidal forces create compression (negative tidal strain), cracks close, resulting in an increase in seismic velocity. Conversely, when tidal forces induce extension (positive tidal strain), cracks open, leading to a decrease in seismic velocity (Fig. 4). The higher density of cracks within fault zones indicates a greater impact of tidal forces on velocity changes in these areas.

The dv/v versus strain sensitivity at diurnal and semidiurnal periods is ∼10^5^ inferred from Fig. 3E and F, consistent with previously reported values [49]. This high sensitivity is reasonable, considering that the number of fractures in the fault fracture zone is roughly ten times greater than in the surrounding wall rock [45]. Additionally, the presence of fractures in the fault fracture zone reduces the overall mechanical strength of the rock mass, making it more susceptible to deformation under tidal forces, which contributes to the enhanced sensitivity of the fault zone to these forces [49]. Furthermore, the dv/v versus strain sensitivity at monthly periods is ∼10^6^ as inferred from Fig. 3C, which is higher than that observed at diurnal and semidiurnal periods. This difference may be due to the nonlinear response of dv/v to tidal strain variations. At lower strain rates, the rate of fracture opening and closing increases, meaning that slower strain changes result in more pronounced fracture opening and closing [48]. Since the tidal strain rate for monthly periods (∼10^−11^/day) is only 0.01 times that of diurnal and semidiurnal periods (∼10^−9^/day), its impact on fracture dynamics is about ten times greater [48], resulting in higher dv/v versus strain sensitivity at monthly periods.

In addition to the increased occurrence of cracks within the fault fracture zone, several other factors may influence the observed dv/v results. First, tidal forces can cause changes in groundwater levels [6], and these water level fluctuations may lead to changes in seismic wave velocity [13]. Furthermore, the higher permeability of fluids within the fault fracture zone could amplify these effects, potentially enhancing the fault zone's response to tidal forces. Second, studies have indicated that the rigidity of fault zones undergoes noticeable temporal variations with periods ranging from 27 to 32 days, which are influenced by the Earth tides [50]. These temporal variations in rigidity could potentially contribute to the observed monthly velocity changes. In this study, we only have 3 months of seismic data. Longer-duration seismic data would be helpful for improving the reliability of the results, particularly in detecting more reliable monthly variations.

Since velocity changes can reflect stress variations, our observations indicate that the stress changes within the Anninghe fault zone are influenced by Earth tides. Tidal forces alter the stress state on fault planes, potentially leading to fault instability and rupture [23,25]. We analyzed the earthquake catalog for the Anninghe fault zone from 2013 to 2020, which recorded a total of 1441 earthquakes with focal depths of less than 15 km [51] (Fig. S8). Our findings reveal a significant increase in earthquake frequency near the lunar perigee compared to other times (Fig. S8B), and suggest a clear daily pattern in the distribution of earthquake occurrences (Fig. S8C). While diurnal changes may largely be attributed to varying human activities during the day and night, the significant uptick in earthquakes around the lunar perigee likely stems from stress responses within the fault zone. These results suggest the presence of stress changes related to Earth tides within the Anninghe fault, which supports our observations of seismic velocity changes. Additionally, this data indicates the potential for earthquakes to be triggered by solid Earth tides within the fault zone [23].

Our observations indicate that, in the absence of earthquakes, stress changes within the shallow fault zone are primarily driven by Earth tides, with this zone exhibiting greater sensitivity to tidal forces than other regions. At greater depths, the influence of environmental factors decreases, potentially amplifying the prevailing influence of tidal forces on the fault's stress state. The stress variations [2] are closely linked to earthquake triggering, suggesting a potential role of tidal modulation in seismic activity. Our work demonstrates that utilizing dense arrays enables effective monitoring of medium variations within narrow fault zones. Prior to major earthquakes, more pronounced stress changes occur within fault zones [10,12]. This method has the potential to assist in monitoring and exploring precursory information related to the occurrence of large earthquakes.

## MATERIALS AND METHODS

### Anninghe array data

Our study area is located within the active segment of the Anninghe fault zone, which extends from Mianning to Xichang in the southeastern margin of the Tibetan Plateau (Fig. 1A). We deployed a dense linear network of 88 seismographs (QS-5A: 5 s–250 Hz effective frequency band, 100 Hz sampling rate, 3C type) along a nearly east-west–oriented line perpendicular to the fault zone (Fig. 1B). The seismographs were spaced ∼50–100 meters apart, forming a linear array. They continuously recorded ground motion from October 2019 to January 2020.

### Estimating seismic velocity change by the wavelet method

We can obtain the empirical Green's function by auto-correlating the ambient noise. Green's function contains information about the structures and elastic properties of the crustal medium [52,53]. By repeating noise interferometry at different times, we estimated Green's functions for consecutive dates (Figs S9A and S10). To determine seismic velocity changes, we used a wavelet method based on wavelet cross-spectrum analysis [18]. This technique focuses on the coda of the reconstructed Green's functions. Coda waves, which are the late arrivals resulting from multiply scattered waves, have longer propagation paths through the medium, making them more sensitive to velocity changes than direct waves [54,55]. This method involves performing cross-spectrum analysis on coda waveforms from different dates to obtain the travel time perturbation (dt) between the two waveforms in the coda window (Fig. S9C). The relative velocity change is the opposite of the travel time perturbation (dv/v = −dt/t). Through this cross-spectrum analysis, we can detect minute perturbations in seismic velocity (dv/v on the order of 10^−4^) [41].

In this study, we initially estimated the daily resolution dv/v for each station. To do this, we derived a reference Green's function by stacking the auto-correlation functions over the entire study period. By analyzing the coda within a 30- to 60-second window of the daily auto-correlation functions and the reference Green's function, we calculated the dv/v within the 1–3 Hz frequency range. Additionally, we tested different coda wave time windows (Fig. S11). The results from these tests were consistent in their temporal and spatial variation characteristics, indicating the reliability of the dv/v results. Time windows ranging from 30 to 60 seconds provided a better signal-to-noise ratio, leading to more stable outcomes. Furthermore, we selected three adjacent stations with high-quality data (Sta64, Sta65, Sta66) and performed cross-correlation calculations for each pair. Using the same processing procedure, we calculated the velocity changes for the three station pairs, averaged the results, and compared them with the velocity changes obtained from the auto-correlation of the three stations. Both methods showed very good consistency (Fig. S12B), indicating the reliability of our results. Additionally, the body waves in the cross-correlation results showed no shift (Fig. S12A), which rules out the influence of clock errors.

Second, we estimated the hourly resolution dv/v for the three group stations (Fig. 1B). For each group, we derived the reference Green's function by stacking the empirical Green's functions obtained from each hour. By employing the wavelet cross-spectrum analysis between the empirical Green's function of each hour and the reference Green's function [18], we were able to determine the seismic wave velocity changes with hourly resolution within the frequency range of 1–2 Hz.

Coda waves are generated by multiple scattering within the medium and are less affected by variations in noise sources [29]. Additionally, we conducted a power spectrum analysis of ambient noise, which revealed no significant or periodic changes (Fig. S13), confirming that there were no significant changes in the noise sources, thus ruling out the influence of source variations on the observed velocity changes.

### Simulations of tidal strain

To simulate the tidal strain at the Anninghe fault, we utilized the PyGTide program [36], which calculates theoretical tidal strain based on inputs of latitude, longitude and time range. Seismic velocity changes calculated using the vertical component of ambient noise, which are dominated by Rayleigh waves, are more sensitive to vertical strain. Therefore, in this study, we focused on vertical tidal strain for our analysis. We then performed spectral analysis on our strain results and extracted the time series of the monthly, diurnal and semidiurnal cycles using the PyGTide program (Fig. S4).

### Standard spectral ratio

To gain a better understanding of the fault zone's subsurface properties and to aid in interpreting velocity changes in its vicinity, we conducted a study using the waveforms of teleseismic earthquakes recorded by the deployed station array. Fault fracture zones in the crust have the ability to trap and amplify seismic waves, leading to intense and prolonged ground shaking at the surface [56]. In this study, we selected 13 teleseismic earthquakes (Table S1) with a good signal-to-noise ratio. We applied the standard spectral ratio (SSR) method to the observed east-west ground motions from the selected local and teleseismic earthquakes. We specifically focused on the east-west components because horizontal ground motions are more significantly affected by local site conditions [44], and the amplification effect is particularly pronounced on the east-west component (Fig. S14). The SSR method necessitates a reference site, typically the bedrock site, from which observed ground motions can be considered input motions for neighboring sites [44]. We selected five bedrock stations as our reference sites [34] (Fig. 1B). For each teleseismic earthquake event, we extracted 20-second waveforms starting from the direct P-wave arrival recorded at all stations and computed the amplitude spectra. Subsequently, we calculated the ratio between the amplitude spectra of each station to the average amplitude spectra of the reference station to derive the standard spectral ratio along the profile line. Finally, we averaged the spectral ratios obtained from the 13 distant seismic events to obtain the final spectral ratio results (Fig. 3B).

## Supplementary Material

nwaf023_Supplemental_File

## Data Availability

The autocorrelation functions used in this study and the velocity change results can be accessed at https://doi.org/10.17632/5zs6c646wb.1.
