# Supplementary material for: Pronounced temporal velocity variations within the fault fracture zone in response to Earth tide modes
Source: Natl Sci Rev. 2025 Jan 22;12(4):nwaf023. doi: 10.1093/nsr/nwaf023 (PMC11904896; doi:10.1093/nsr/nwaf023)
Supplement: nwaf023_Supplemental_File [file nwaf023_supplemental_file.docx]

Supplementary Materials for

**Pronounced temporal velocity variations within the fault fracture zone in response to Earth tide modes**

Tenghui Sun *et al.*

*Corresponding author. Huajian Yao, hjyao@ustc.edu.cn

**This PDF file includes:**

Text S1

Figs. S1 to S14

Table S1

Supplementary References

**Text S1: Estimation of the energy velocity (𝑐) and mean free path (𝑙)**

To determine the composition of the coda waves, it is necessary to estimate the energy velocity (𝑐) and mean free path (𝑙) in the study area [1]. The energy velocity 𝑐, which reflects the celerity at which the seismic energy is transported, can be approximated as: 1/𝑐 = 0.77/v_s_ +0.23/v_p_ [1]. In our study area, with v_s_ ≈ 3 km/s and v_p_ ≈ 5 km/s [2], the calculated energy velocity (𝑐) is approximately 3.4 km/s. The mean free path (𝑙) is related to the scattering attenuation and can be expressed as: Q_s_= 2πf𝑙/𝑐 [3]. For this study, with f = 1.5 Hz and Q_s_ ≈ 200 [4], the mean free path (𝑙) is estimated to be approximately 72 km.

**Reference:**

1. Obermann A, Planès T and Larose E *et al.* Depth sensitivity of seismic coda waves to velocity perturbations in an elastic heterogeneous medium. *Geophys J Int* 2013; **194**: 372–82.

2. Shao X, Yao H and Liu Y *et al.* Shallow crustal velocity structures revealed by active source tomography and fault activities of the Mianning–Xichang segment of the Anninghe fault zone, Southwest China. *Earth Planet Phys* 2022; **6**: 204–12.

3. Obermann A, Planès T and Larose E *et al.* Imaging preeruptive and coeruptive structural and mechanical changes of a volcano with ambient seismic noise. *J Geophys Res Solid Earth* 2013; **118**: 6285–94.

4. Zhou L, Zhao C and Xiu J *et al*. Tomography of Q_Lg_ in Sichuan-Yunnan Zone. *Chin J Geophys* (in Chinese) 2008; **51**: 1745-1752.


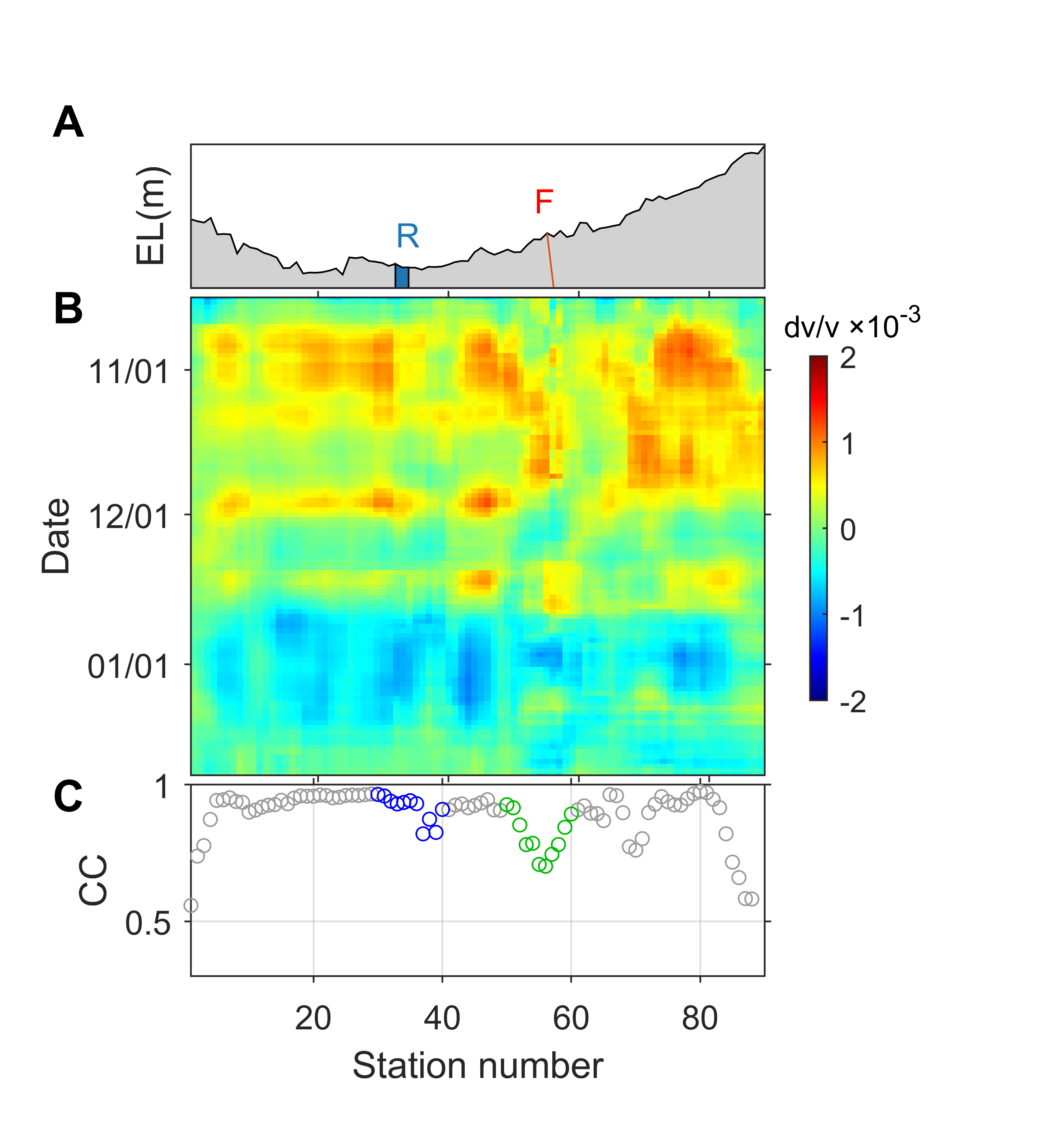


**Figure S1. The relative seismic velocity changes (dv/v) at daily resolution in the frequency band of 2-3 Hz.** (**A**) The spatial distribution of river and fault zone. The blue area represents the Anninghe River (‘R’) and the red line represents the Anninghe Fault (‘F’). (**B**) The dv/v at daily resolution of each station. (**C**) The correlation coefficient between the dv/v of each station and the average dv/v of all stations. The blue dots represent the correlation coefficients of stations located beside the river, while the green dots correspond to the blue triangles in Figure 1B, which represent the correlation coefficients of stations near the fault zone.


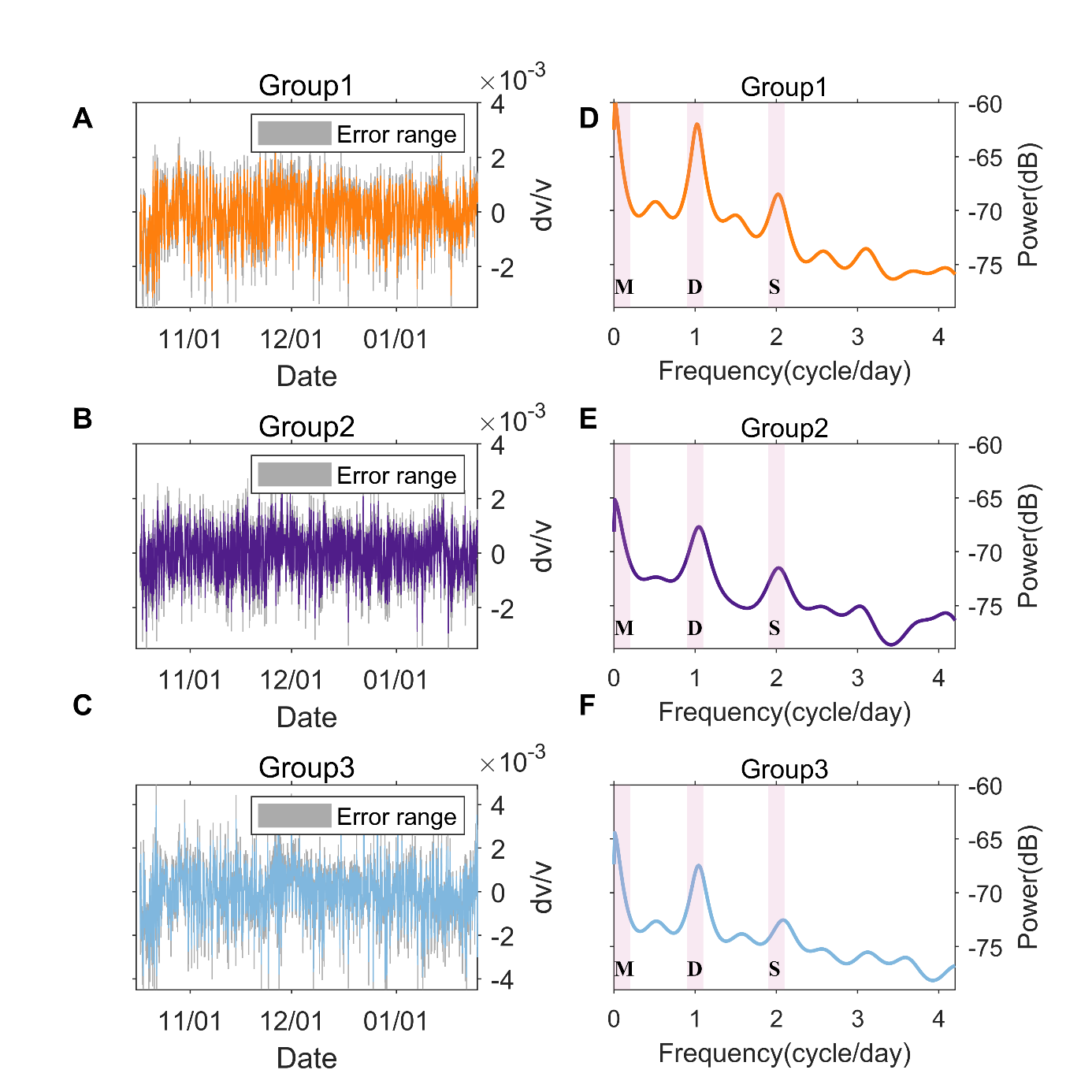


**Figure S2. The hourly resolution dv/v.** (A-C) The hourly resolution dv/v curves for Group 1, Group 2, and Group 3 stations, respectively. (D-F) The spectrum of dv/v for Group 1, Group 2, and Group 3 stations, respectively (‘M’ represents monthly, ‘D’ represents diurnal, ‘S’ represents semidiurnal).

**Figure S3.** **Depth-dependent sensitivity kernels of Rayleigh waves.** (**A**) Velocity profile for the study region. (**B**) Sensitivity kernels of the different frequency bands.


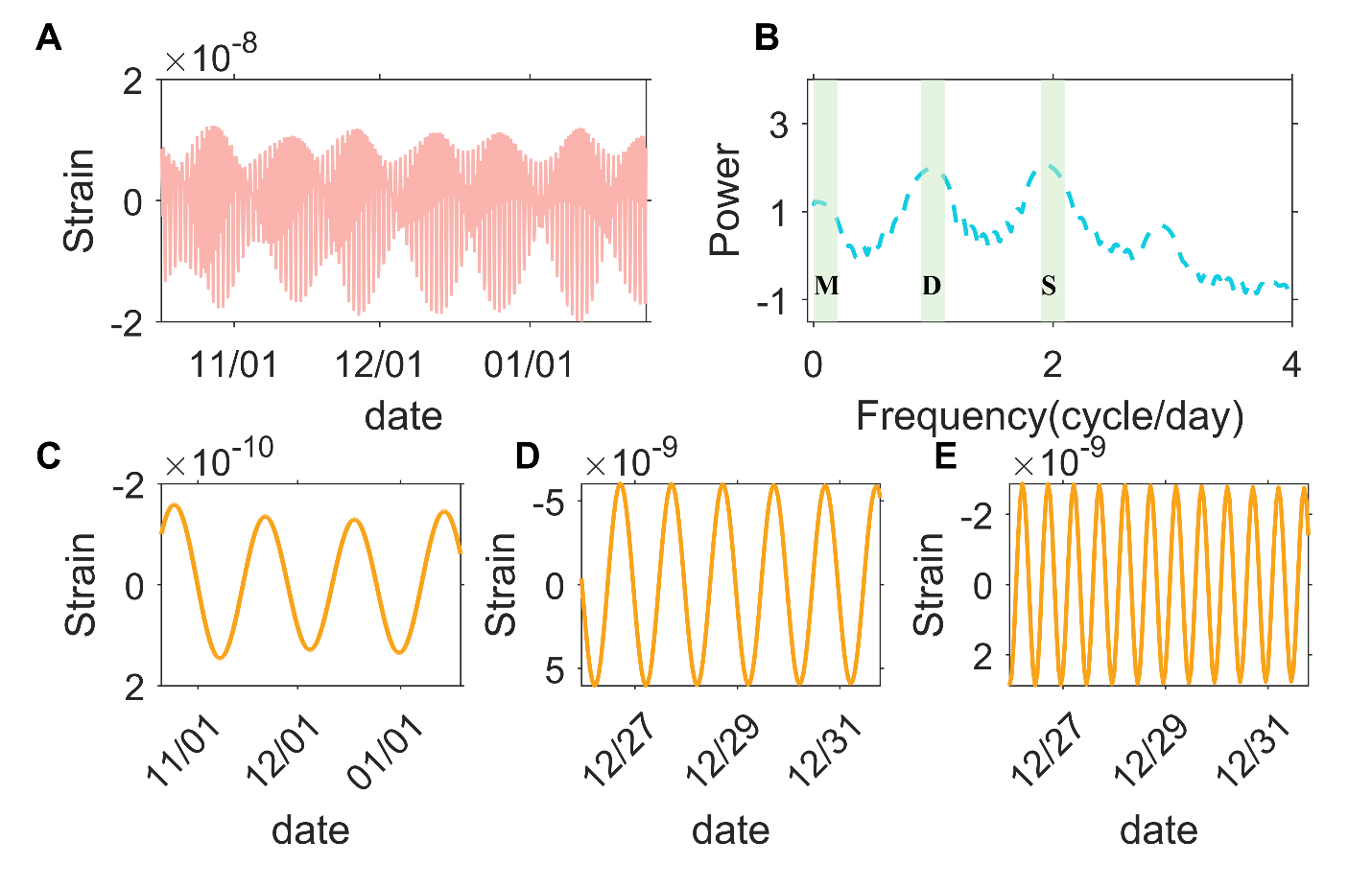


**Figure S4. Tidal strain in the vertical component in the study area modeled by PyGTide.** (A) Time series of vertical tidal strain. (B) Tidal strain spectrum (‘M’ represents monthly, ‘D’ represents diurnal, ‘S’ represents semidiurnal). (C) Monthly tidal strain time series. (D) Diurnal tidal strain time series. (E) Semidiurnal tidal strain time series. Positive values indicate expansion, while negative values indicate compression.


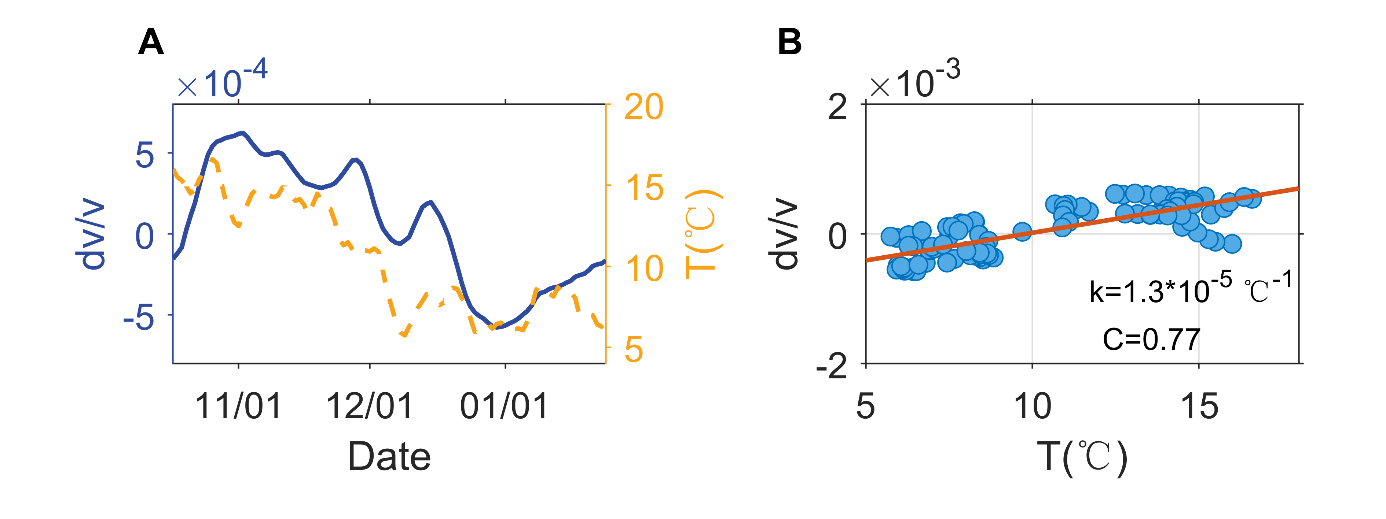


**Figure S5. Comparison of temperature changes and dv/v influenced by environmental factors in the 2-3 Hz frequency range.** (**A**) The curves of temperature (yellow dashed line) and dv/v (blue line). (**B**) Linear fitting of temperature and dv/v in the 2-3 Hz frequency range. ‘k’ is the slope of the fitted curve, and ‘C’ is the correlation coefficient.


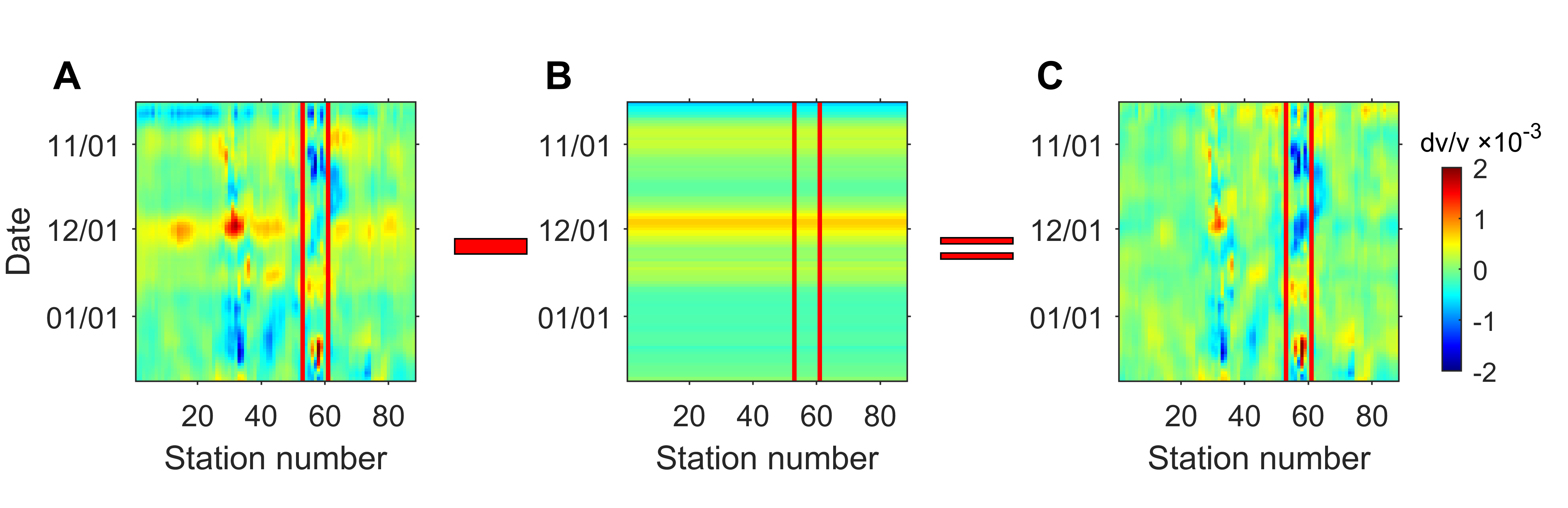


**Figure S6. Mitigation of the influence of environmental factors.** (**A**) The daily dv/v resolution of each station in the frequency band of 1-2 Hz. (**B**) The impact of environmental factors on dv/v, determined by calculating the average dv/v from stations with a strong correlation (correlation coefficient > 0.75, as shown in Fig. 2C). (**C**) The dv/v after removing the influence of environmental factors.


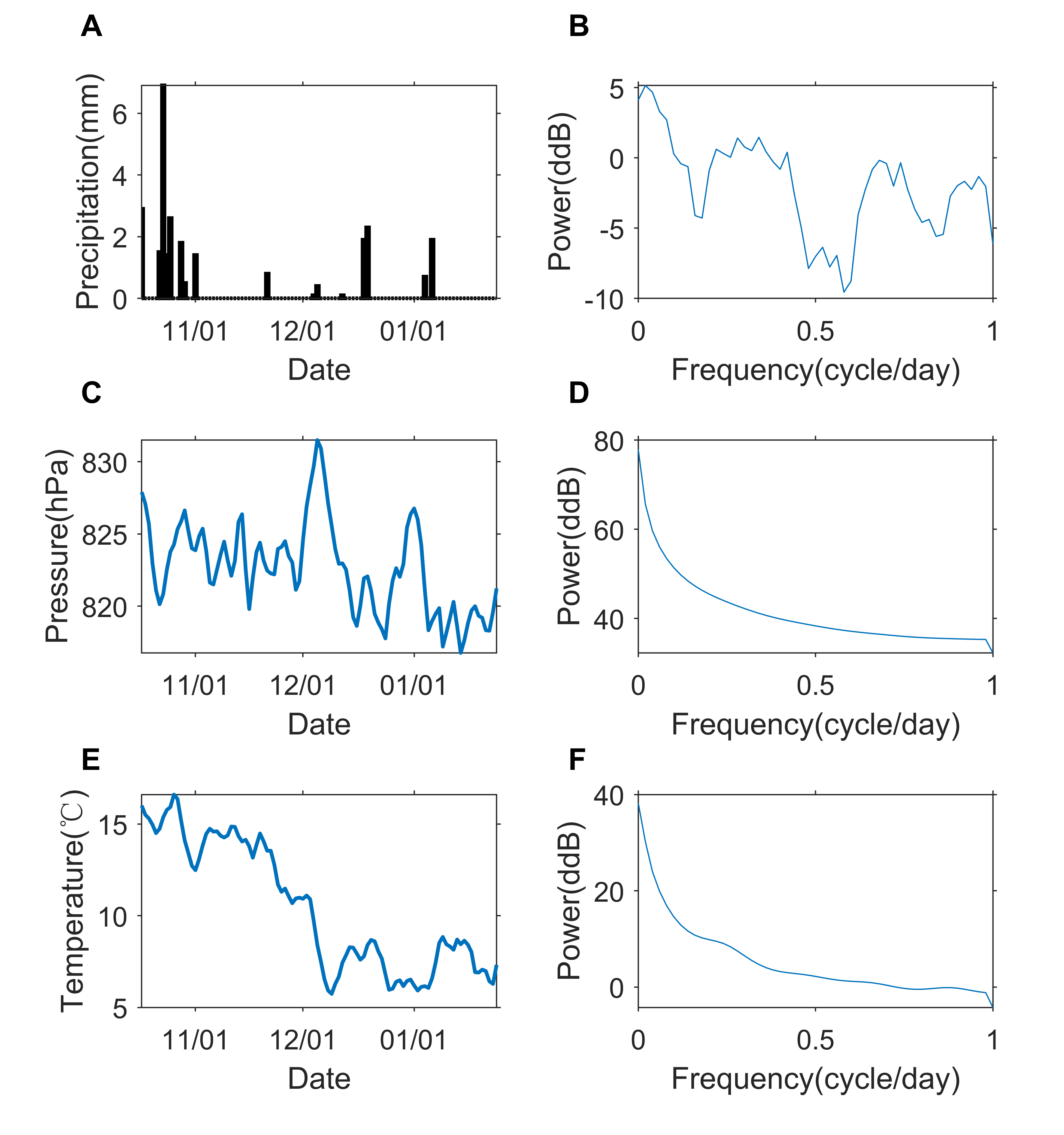


**Figure S7. Environmental factors: (A, B) Precipitation and its spectrum, (C, D) Barometric pressure and its spectrum, (E, F) Temperature and its spectrum.**


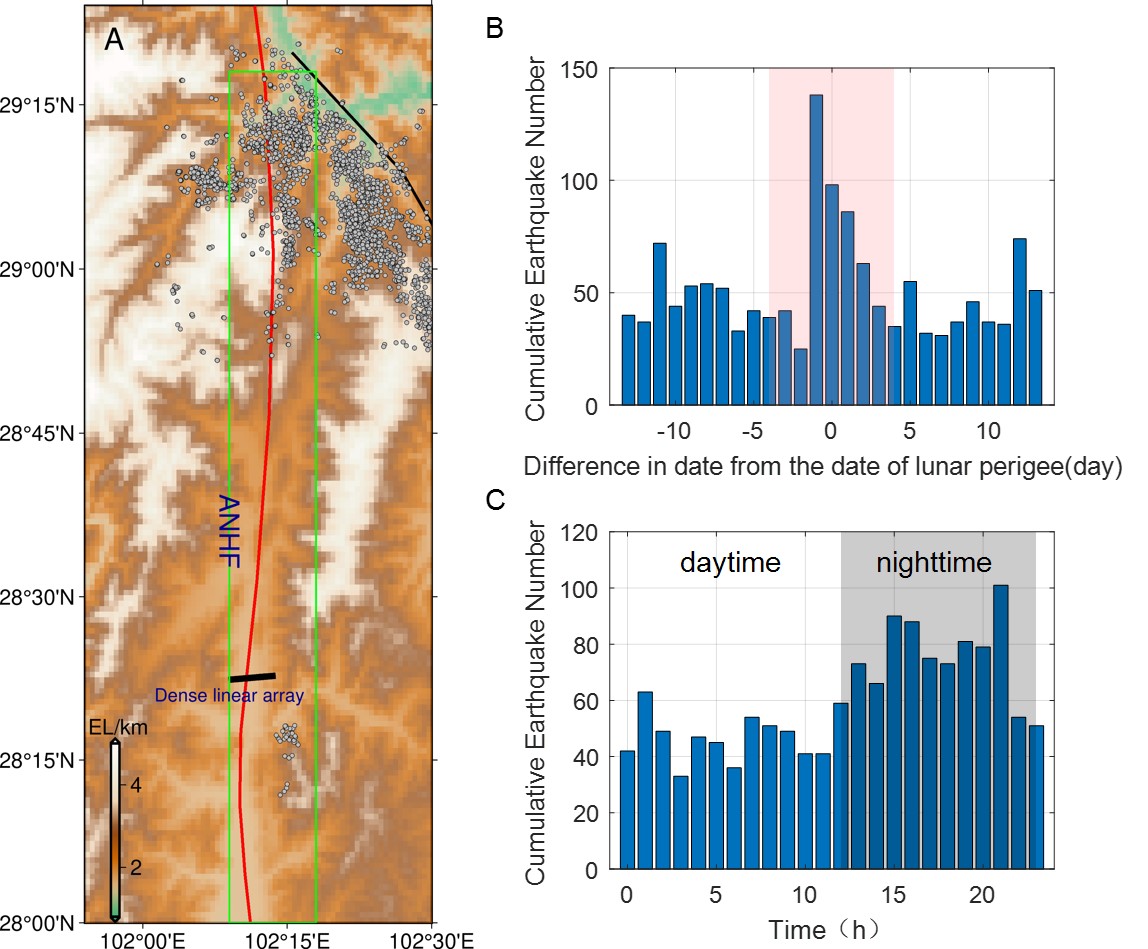


**Figure S8. The spatiotemporal distribution of earthquakes in the Anninghe Fault Zone. (A)** Earthquakes near the Anninghe fault from 2013 to 2020. The green box represents our statistical range. **(B)** The cumulative number of earthquakes on different days relative to the date of the lunar perigee. The shaded region indicates dates that fall within four days of the perigee. **(C)** Hour of the day seismicity frequency.


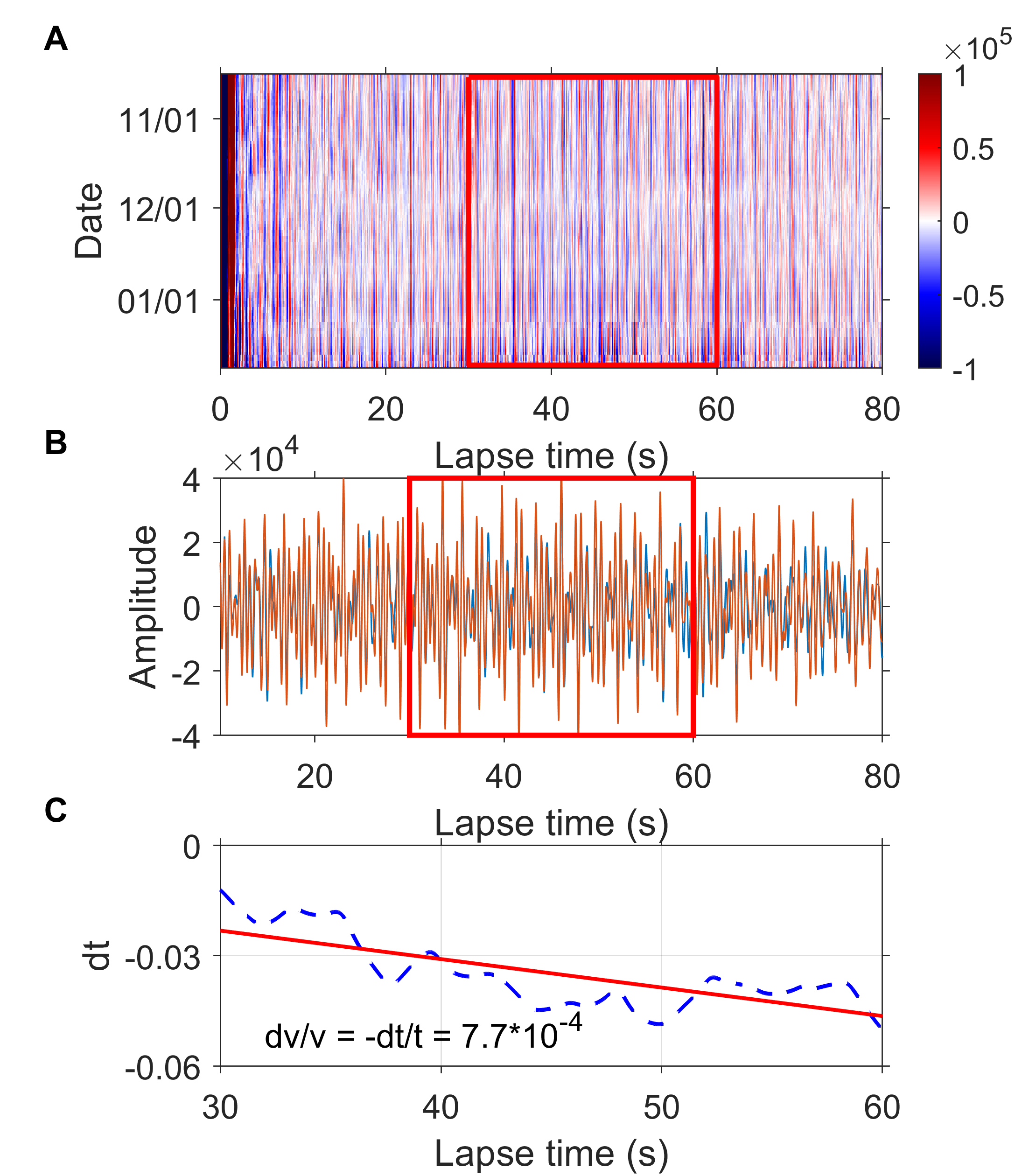


**Figure S9.** **Daily reconstructed Green’s function (Example: Sta15).** (**A**) Map of the reconstructed Green's function. The red rectangle indicates the coda wave window used. (**B**) Comparison of the autocorrelation function (red curve), and reference Green’s function (blue curve). The red rectangle indicates the coda wave window used. (**C**) The travel time perturbation in the coda window calculated by the wavelet method. The blue dashed line represents the travel time perturbation, and the red solid line is the fitted straight line with a slope of dt/t. The relative velocity change dv/v is given by dv/v = -dt/t.


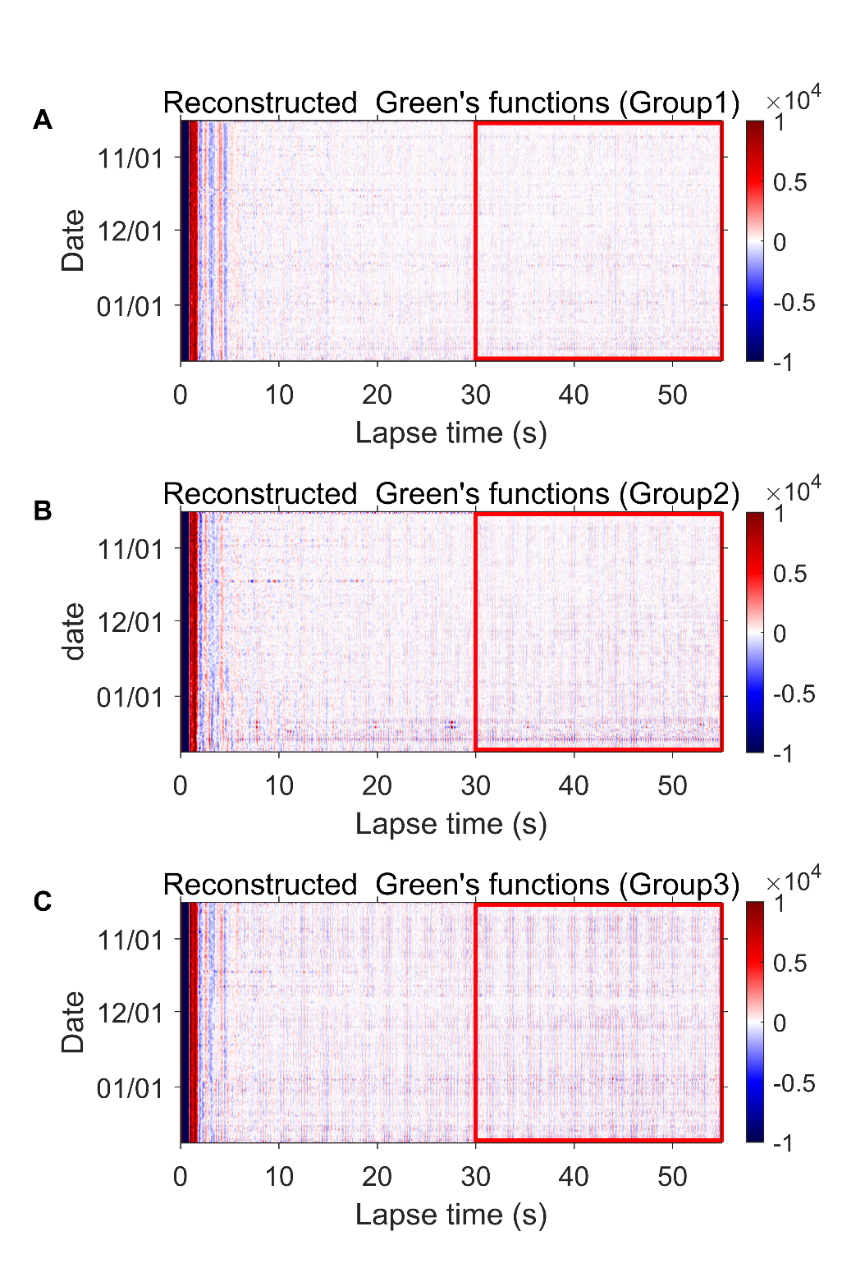


**Figure S10.** **Hourly reconstructed Green’s function.** (**A**) The reconstructed Green’s function of Group 1. (**B**) The reconstructed Green’s function of Group 2. (**C**) The reconstructed Green’s function of Group 3. The red rectangles indicate the coda wave window used.


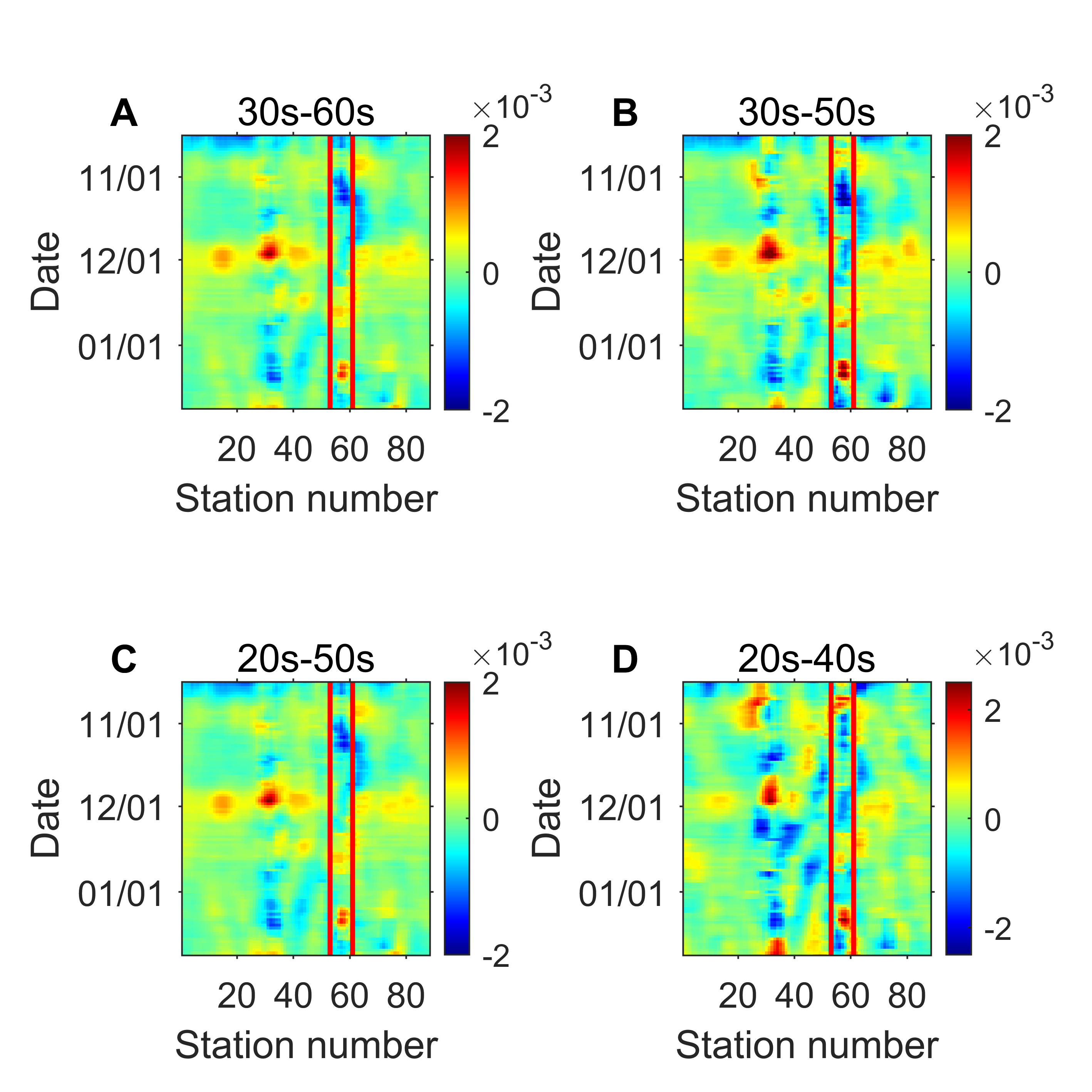


**Figure S11. Results of dv/v calculated from different coda windows**.


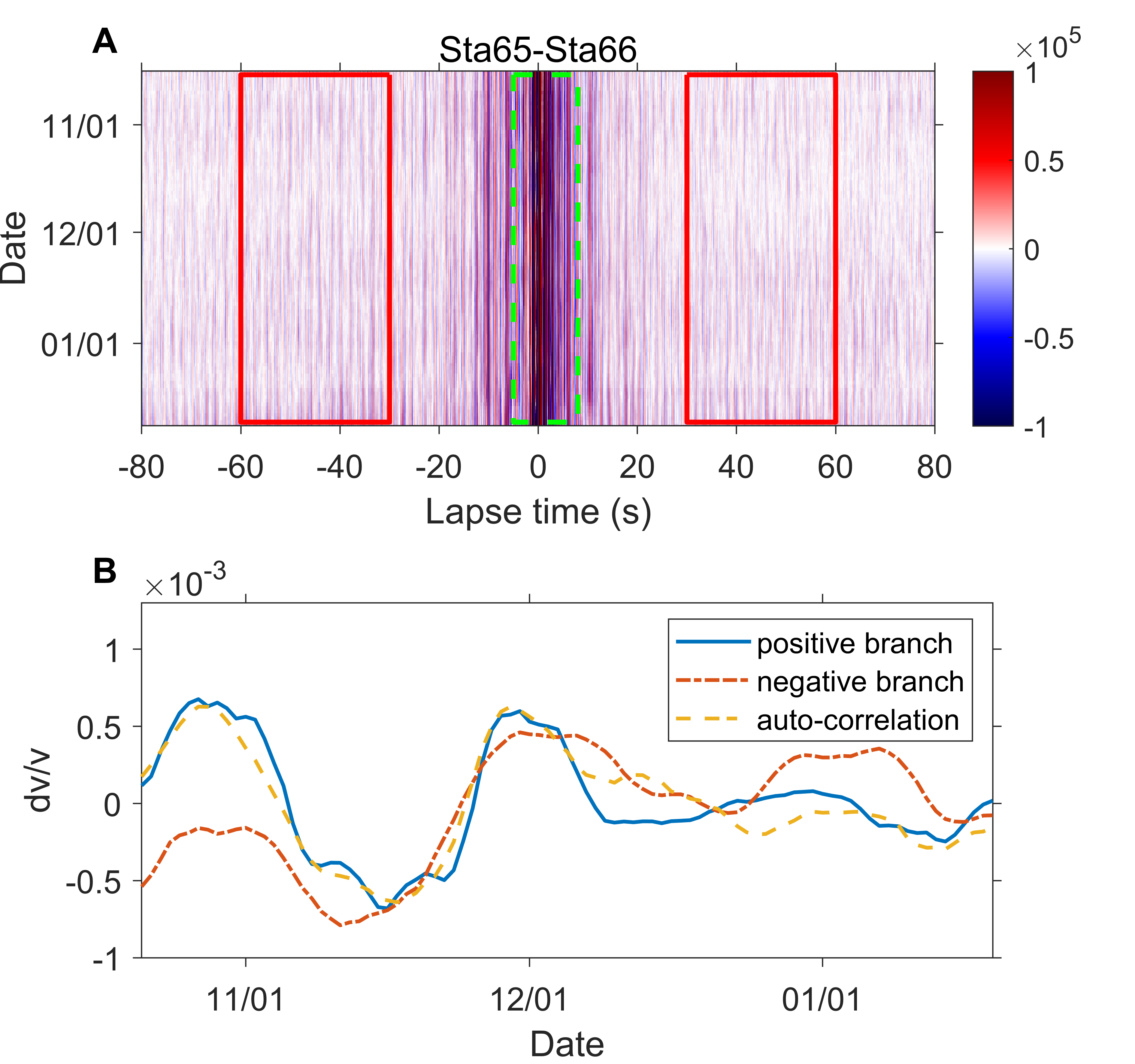


**Figure S12. Comparison of dv/v calculated from auto-correlation and cross-correlation.** (**A**) The cross-correlation function of Sta65 and Sta66. The red rectangles indicate the coda wave window used. The green dashed rectangle indicates the body wave window. (**B**) The blue line represents the dv/v from the positive branches of cross-correlation, the red dashed line represents the dv/v from the negative branches of cross-correlation, and the yellow dashed line represents the dv/v from auto-correlation.


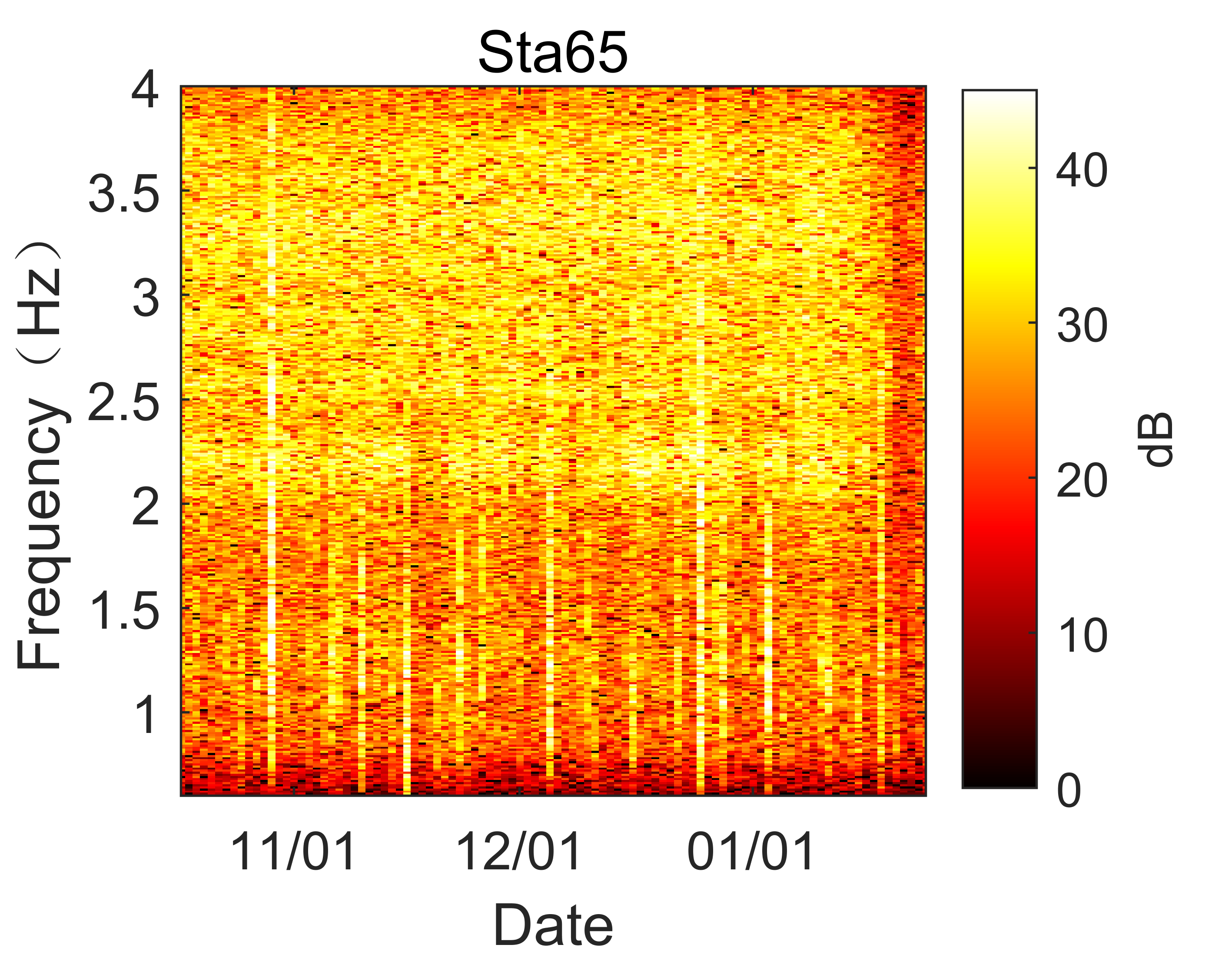


**Figure S13.** The power spectrum of the seismic ambient noise.


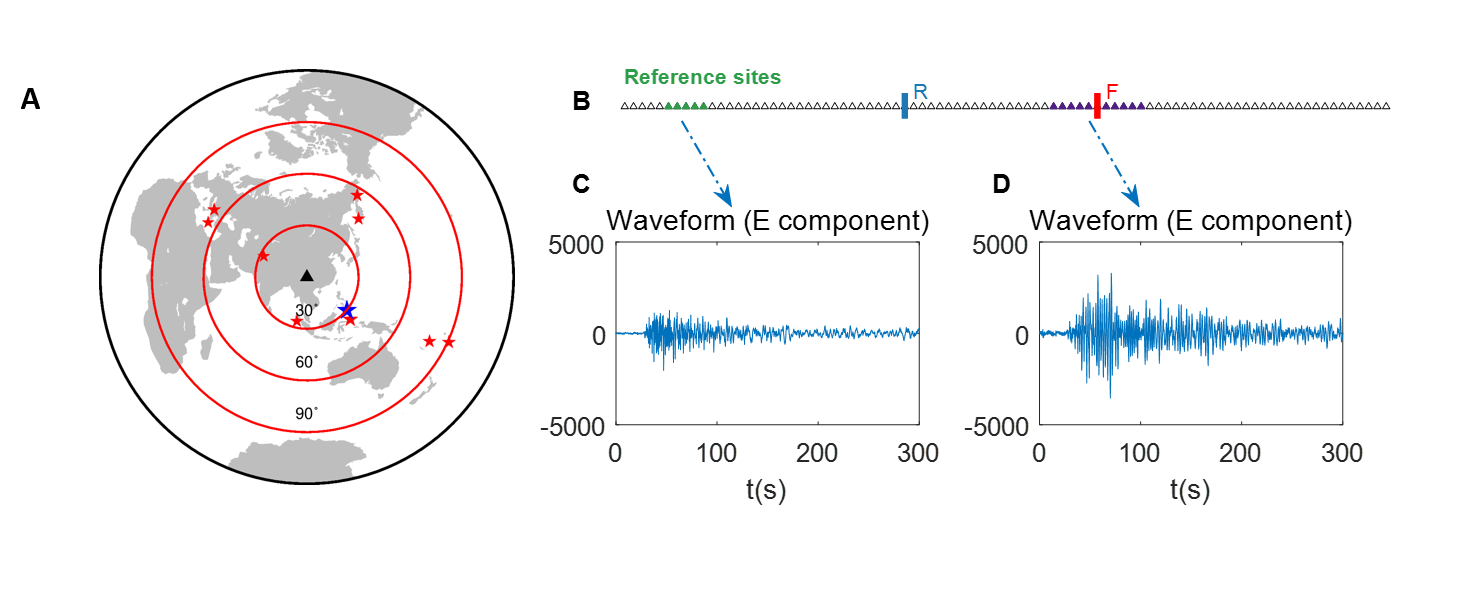


**Figure S14. Spatial distribution of the teleseismic earthquakes (circles) and the waveforms.**

**(A)** Spatial distribution of the teleseismic earthquakes. Black triangle represents the dense array and the stars represent the teleseismic earthquakes. Information about all earthquakes analyzed in this study is detailed in Table 1. (**B**) The location of the reference sites. (**C)** Waveform of one teleseismic earthquake (indicated by the blue star in A) recorded at one station in the reference site. (**D**) Waveform of one teleseismic earthquake (indicated by the blue star in A) recorded at one station in the fault zone.

**Table S1 Selected Earthquakes**

| ID | Origin time | Longitude (°) | Latitude (°) | Depth (km) | Magnitude (Mw) |
| --- | --- | --- | --- | --- | --- |
| T1 | 2020-01-09 08:38:08 | 171.06 | 62.36 | 10 | 6.4 |
| T2 | 2020-01-07 06:05:19 | 96.36 | 2.35 | 17 | 6.3 |
| T3 | 2019-12-20 11:39:52 | 70.46 | 36.54 | 212 | 6.1 |
| T4 | 2019-12-15 06:11:51 | 125.17 | 6.70 | 18 | 6.8 |
| T5 | 2019-11-27 07:23:42 | 23.23 | 35.72 | 69 | 6 |
| T6 | 2019-11-26 02:54:12 | 19.53 | 41.51 | 22 | 6.4 |
| T7 | 2019-11-20 08:26:08 | 153.68 | 53.13 | 496 | 6.3 |
| T8 | 2019-11-14 21:12:54 | 126.41 | 1.54 | 23 | 6 |
| T9 | 2019-11-14 16:17:40 | 126.42 | 1.62 | 33 | 7.1 |
| T10 | 2019-11-08 10:44:44 | -179.51 | -21.94 | 577 | 6.5 |
| T11 | 2019-10-31 01:11:19 | 125.18 | 6.91 | 10 | 6.5 |
| T12 | 2019-10-29 01:04:43 | 125.01 | 6.76 | 15 | 6.6 |
| T13 | 2019-10-21 02:52:29 | 169.49 | -19.02 | 231 | 6.4 |
